# Supplementary material for: Cytosolic concentrations of actin binding proteins and the implications for in vivo F-actin turnover
Source: J Cell Biol. 2023 Oct 6;222(12):e202306036. doi: 10.1083/jcb.202306036 (PMC10558290; doi:10.1083/jcb.202306036)
Supplement: Table S1 — shows antibodies used for Western blots and the yeast actin binding proteins purified for generating standard curves. [file JCB_202306036_TableS1.docx]

Supplementary Table 1. Antibodies used for Western blots and the yeast actin binding proteins purified for generating standard curves.

| **Protein name** | **Primary**  **antibody source** | **Antibody reference** | **Primary**  **antibody dilution** | **Protein**  **expression system** | **Protein**  **purification procedure** |
| --- | --- | --- | --- | --- | --- |
| Abp1 | chicken | This study | 1:10,000 | *S. cerevisiae* | Goode et al., 2001 |
| Act1 | chicken | This study | 1:20,000 | *S. cerevisiae* | Goode, 2002 |
| Aip1 | chicken | This study | 1:500a | *S. cerevisiae* | Rodal et al., 1999 |
| Arp2 | rabbit | Moreau et al., 1996 | 1:1,000 | *S. cerevisiae* | Goode, 2002 |
| Cap1/ Cap2 | chicken | This study | 1:8,000 | *S. cerevisiae* | Amatruda et al., 1992 |
| Cof1 | chicken | This study | 1:5,000 | *E. coli* | Lappalainen et al.,  1997 |
| Crn1 | mouse | Goode et al., 1999 | 1:1,000 | *E. coli* | Goode et al., 1999 |
| Srv2 | chicken | This study | 1:7,000 | *S. cerevisiae* | Balcer et al., 2003 |
| Sac6 | chicken | This study | 1:10,000 | *S. cerevisiae* | Goodman et al., 2003 |
| Scp1 | rabbit | Goodman et al., 2003 | 1:2,000a | *E. coli* | Goodman et al., 2003 |
| Tpm1 | chicken | This study | 1:500a | *S. cerevisiae* | Liu et al., 1989 |
| Twf1 | rabbit | This study | 1:1,000 | *E. coli* | Goode et al., 1998 |

**a** These antibodies were affinity purified.
