# Supplementary material for: Cytosolic concentrations of actin binding proteins and the implications for in vivo F-actin turnover
Source: J Cell Biol. 2023 Oct 6;222(12):e202306036. doi: 10.1083/jcb.202306036 (PMC10558290; doi:10.1083/jcb.202306036)
Supplement: Table S2 — shows comparison of the cytosolic fractions of five ABPs in S. pombe (Sirotkin et al., 2010) versus S. cerevisiae (this study). [file JCB_202306036_TableS2.docx]

Supplementary Table 2. Comparison of the cytosolic fractions of five ABPs in *S. Pombe* (Sirotkin et al., 2010) vs. *S. cerevisiae* (this study).

*S. cerevisiae+ S. pombe**

| **Name** | **% free in cytosol** | **Cytosolic concentration (µM)** | **% free in cytosol** | **Cytosolic concentration (µM)** |
| --- | --- | --- | --- | --- |
| Arp2/3 complex | 81% | 0.80 | 78% | 2.3 |
| Fimbrin | 80% | 1.0 | 68% | 3.7 |
| Capping protein | 67% | 0.90 | 66% | 0.8 |
| Coronin | 63% | 0.90 | 73% | 3.1 |
| Twinfilin | 71% | 0.80 | 65% | 1.4 |

+Data from this study. See Table 1 for more details.

*Data from Sirotkin *et al*., 2010.
