# Supplementary material for: Cytosolic concentrations of actin binding proteins and the implications for in vivo F-actin turnover
Source: J Cell Biol. 2023 Oct 6;222(12):e202306036. doi: 10.1083/jcb.202306036 (PMC10558290; doi:10.1083/jcb.202306036)
Supplement: Table S3 — shows S. cerevisiae strains used in this study. [file JCB_202306036_TableS3.docx]

Supplementary Table 3. *S. cerevisiae* strains used in this study

| **Strain** | **Genotype** | **Strain background** | **Source** |
| --- | --- | --- | --- |
| BGY10 | **MAT**a, *his3-11; ura3-52; leu2-3, 112; ade2-1; trp1-1; psi+* | W303 | Goode et al., 1999 |
| BGY12 | **MAT**α*, his3-11; ura3-52; leu2-3, 112; ade2-1; trp1-1; psi+* | W303 | Goode et al., 1999 |
| BGY4360 | **MAT**a, *AIP-GFP::HIS3 his3Δ1 leu2Δ0 met15Δ0 ura3Δ0* | BY4741 | ResGen^a^ |
| BGY4361 | **MAT**a, *TWF1-GFP::HIS3 his3Δ1 leu2Δ0 met15Δ0 ura3Δ0* | BY4741 | ResGen^a^ |
| BGY4362 | **MAT**a, *ABP1-GFP::HIS3 his3Δ1 leu2Δ0 met15Δ0 ura3Δ0* | BY4741 | ResGen^a^ |
| BGY4363 | **MAT**a, *CAP1-GFP::HIS3 his3Δ1 leu2Δ0 met15Δ0 ura3Δ0* | BY4741 | ResGen^a^ |
| BGY4364 | **MAT**a, *CRN1-GFP::HIS3 his3Δ1 leu2Δ0 met15Δ0 ura3Δ0* | BY4741 | ResGen^a^ |
| BGY4365 | **MAT**a, *ARC15-GFP::HIS3 his3Δ1 leu2Δ0 met15Δ0 ura3Δ0* | BY4741 | ResGen^a^ |
| BGY4366 | **MAT**a, *SAC6-GFP::HIS3 his3Δ1 leu2Δ0 met15Δ0 ura3Δ0* | BY4741 | ResGen^a^ |
| BGY4367 | **MAT**a, *SRV2-GFP::HIS3 his3Δ1 leu2Δ0 met15Δ0 ura3Δ0* | BY4741 | ResGen^a^ |
| BGY4368 | **MAT**a, *ARP2-GFP::HIS3 his3Δ1 leu2Δ0 met15Δ0 ura3Δ0* | BY4741 | ResGen^a^ |
| BGY4369 | **MAT**a, *SCP1-GFP::HIS3 his3Δ1 leu2Δ0 met15Δ0 ura3Δ0* | BY4741 | ResGen^a^ |
| BGY4370 | **MAT**a, *CAP2-GFP::HIS3 his3Δ1 leu2Δ0 met15Δ0 ura3Δ0* | BY4741 | ResGen^a^ |
| BGY4371 | **MAT**a, *PFY1-GFP::HIS3 his3Δ1 leu2Δ0 met15Δ0 ura3Δ0* | BY4741 | ResGen^a^ |
| BGY4372 | **MAT**a, *AIP1-GFP::HIS3 ARC15-mSCARLET::KANR his3Δ1 leu2Δ0 met15Δ0 ura3Δ0* | BY4741 | This study |
| BGY4373 | **MAT**a, *TWF1-GFP::HIS3 ARC15-mSCARLET::KANR his3Δ1 leu2Δ0 met15Δ0 ura3Δ0* | BY4741 | This study |
| BGY4374 | **MAT**a, *ABP1-GFP::HIS3 ARC15-mSCARLET::KANR his3Δ1 leu2Δ0 met15Δ0 ura3Δ0* | BY4741 | This study |
| BGY4375 | **MAT**a, *ABP140-GFP::HIS3 ARC15-mSCARLET::KANR his3Δ1 leu2Δ0 met15Δ0 ura3Δ0* | BY4741 | This study |
| BGY4313 | **MAT**a, *CAP1-GFP::HIS3 ARC15-mSCARLET::KANR his3Δ1 leu2Δ0 met15Δ0 ura3Δ0* | BY4741 | This study |
| BGY4376 | **MAT**a, *CRN1-GFP::HIS3 ARC15-mSCARLET::KANR his3Δ1 leu2Δ0 met15Δ0 ura3Δ0* | BY4741 | This study |
| BGY4358 | **MAT**a, *ARC15-mSCARLET::KANR his3Δ1 leu2Δ0 met15Δ0 ura3Δ0* | BY4741 | This study |
| BGY4377 | **MAT**a, *SAC6-GFP::HIS3 ARC15-mSCARLET::KANR his3Δ1 leu2Δ0 met15Δ0 ura3Δ0* | BY4741 | This study |
| BGY4359 | **MAT**a, *SRV2-GFP::HIS3 ARC15-mSCARLET::KANR his3Δ1 leu2Δ0 met15Δ0 ura3Δ0* | BY4741 | This study |
| BGY4378 | **MAT**a, *ARP2-GFP::HIS3 ARC15-mSCARLET::KANR his3Δ1 leu2Δ0 met15Δ0 ura3Δ0* | BY4741 | This study |
| BGY4379 | **MAT**a, *SCP1-GFP::HIS3 ARC15-mSCARLET::KANR his3Δ1 leu2Δ0 met15Δ0 ura3Δ0* | BY4741 | This study |
| BGY4314 | **MAT**a, *CAP2-GFP::HIS3 ARC15-mSCARLET::KANR his3Δ1 leu2Δ0 met15Δ0 ura3Δ0* | BY4741 | This study |
| BGY4380 | **MAT**α***,*** *PFY1-GFP::HIS3 ARC15-mSCARLET::KANR his3Δ1 leu2Δ0 met15Δ0 ura3Δ0* | BY4741 | This study |
| BGY4381 | **MAT**a*, his3-11, 15; ura3-52; leu2-3, 112; ade2-1; trp1-1; psi+; mNeon-tpm1::LEU2* | W303 | This study |
| BGY4382 | **MAT**α*, his3-11, 15; ura3-52; leu2-3, 112; ade2-1; trp1-1; psi+; mNeon-tpm2::TRP1* | W303 | This study |
| BGY4383 | **MAT**α*, his3-11, 15; ura3-52; leu2-3, 112; ade2-1; trp1-1; psi+; abp140-mNeon::TRP1* | W303 | This study |

aInvitrogen, Thermo Fisher, Waltham, MA
